# The Role of Testing and Vaccination in Mediating Social Vulnerability and COVID-19 Prevalence in Southern Nevada

**DOI:** 10.3390/ijerph22070980

**Published:** 2025-06-21

**Authors:** Andrea Lopez, Lung-Chang Chien, L.-W. Antony Chen, Courtney Coughenour, Erika Marquez, Szu-Ping Lee

**Affiliations:** 1 Department of Epidemiology and Biostatistics, School of Public Health, University of Nevada, Las Vegas, NV 89110, USA; lopeza41@unlv.nevada.edu; 2 Department of Environmental and Occupational Health, School of Public Health, University of Nevada, Las Vegas, NV 89110, USA; antony.chen@unlv.edu (L.-W.A.C.); courtney.coughenour@unlv.edu (C.C.); erika.marquez@unlv.edu (E.M.); 3 Department of Physical Therapy, School of Integrated Health Sciences, University of Nevada, Las Vegas, NV 89110, USA; szu-ping.lee@unlv.edu

**Keywords:** COVID-19, prevalence rate, testing rate, vaccination rate, serial mediation, social vulnerability

## Abstract

The COVID-19 pandemic is a catastrophic event highlighting numerous health disparities. The social vulnerability index (SVI) has been widely utilized in COVID-19 research to assess vulnerable communities and to examine how social determinants influence various COVID-19 outcomes. This population-based study aims to determine whether COVID-19 testing and vaccination rates mediate the relationship between the SVI and COVID-19 prevalence. Mediation analysis was conducted using data from 535 census tracts in Clark County, Nevada. Findings indicate that COVID-19 testing rates were lower in areas with high SVI scores, potentially leading to more undetected cases. Moreover, COVID-19 testing, full vaccination, and follow-up vaccination rates significantly mediated the relationship between SVI and COVID-19 prevalence. These results suggest that greater location-based social vulnerability is associated with a sequential pathway of reduced testing and vaccination rates, contributing to underreported COVID-19 cases.

## 1. Introduction

In May 2020, four months after the first COVID-19 case in the United States (U.S.), nearly 100,000 people died, predominantly in socially vulnerable communities [1]. Approximately 20% of U.S. residents are considered socially vulnerable, facing increased health risks during crises due to inequity in access to healthcare, transportation, education, and financial stability [2]. The Centers for Disease Control and Prevention (CDC) developed a measure called the social vulnerability index (SVI) to classify census tracts based on socioeconomic status, household composition, racial and ethnic minority status, and housing type or transportation [3]. Originally intended for disaster resource allocation [4], the SVI was later applied to adverse public health events. However, fewer than one-third of jurisdictions utilized it to identify areas needing support during the COVID-19 pandemic [5]. The SVI’s wide-ranging utility was demonstrated throughout the COVID-19 pandemic, as factors such as age, low health insurance coverage, and language barriers were associated with infection risk [6,7]. These findings underscore the importance of understanding the role of SVI in disease control.

Research has demonstrated that high SVI scores were associated with higher COVID-19 prevalence, particularly among low-income individuals and those in manual labor occupations [8,9,10]. Vulnerable populations, such as those over 65, under 18, with disabilities, and single-parent households in high SVI areas, exhibited elevated infection risks [10,11,12]. Infections were also more prevalent among Black Americans and Hispanics than among Whites, largely due to financial hardships [13,14,15]. Additionally, crowded housing, rural residency, and lack of transportation further increased the risk of COVID-19 exposure [16,17,18].

Research shows that areas with higher SVI scores often had lower COVID-19 testing rates, indicating social vulnerabilities may hinder access and utilization of essential health services during crises [6,7]. Conversely, a study found no significant correlation between these variables in Alabama, suggesting that local contextual factors may be crucial in determining testing accessibility and uptake [19]. Overall, low testing rates are frequently associated with social vulnerabilities, including older age, lack of insurance, overcrowding, and limited English proficiency, compounding challenges for at-risk populations [20]. Consequently, these factors exacerbate vulnerable populations’ challenges in seeking timely testing.

Regarding vaccination rates, studies show that social determinants, such as lack of health insurance, low education levels, and lower income, which are linked to higher SVI scores, correlated with lower vaccination rates [21,22,23]. Interestingly, unemployed individuals reported higher vaccination rates, while those in blue-collar jobs often exhibited lower vaccination rates due to mistrust in government and healthcare systems [24,25]. Older adults (65+) were more likely to be vaccinated, but individuals with disabilities in high-SVI areas showed no significant association with vaccination uptake [14,26]. Additionally, Blacks and Hispanics in high-SVI areas were vaccinated at higher rates than Whites, despite accessibility challenges [15,27,28]. Rural residents consistently reported lower vaccination rates than their urban counterparts [29]. These findings illustrate complex social determinants influencing vaccination access and uptake.

Understanding the interplay between COVID-19 testing and vaccination is crucial to public health strategies for disease control. Early in the pandemic in the U.S., the human development index, similar to the SVI, was associated with COVID-19 testing rates and prevalence [30,31]. Minority populations exhibited higher testing and positive rates, while uninsured individuals in high-prevalence areas often lacked access to testing [32,33]. The introduction of at-home testing kits in 2022 contributed to underreported testing, despite rising prevalence [34,35]. Conversely, vaccination rates showed an inverse relationship with prevalence and testing, as fully vaccinated individuals, especially those who received boosters, were less likely to seek testing [36,37]. As demonstrated in the literature, ongoing research is needed to address these disparities and guide public health policies.

Given the interconnected associations between the SVI, COVID-19 testing and vaccination rates, and prevalence, there appears to be a potential mediating relationship among them that warrants further investigation. Therefore, in this study, we hypothesize that the SVI is positively associated with COVID-19 prevalence, mediated by COVID-19 testing or vaccination rates. Specifically, this research addresses the following questions: (1) Does the COVID-19 testing rate mediate the positive relationship between the SVI and COVID-19 prevalence? (2) Do the COVID-19 full or follow-up vaccination rates mediate the positive relationship between the SVI and COVID-19 prevalence? (3) Do the COVID-19 testing and vaccination rates simultaneously mediate the positive relationship between the SVI and COVID-19 prevalence? The ultimate goal is to investigate both the direct and indirect effects of the SVI on COVID-19 prevalence by modeling a sequence in which the SVI influences the testing rate, which in turn affects full and follow-up vaccination rates. This sequence reflects the progression of testing and vaccination rollout in the U.S. This study aims to provide health agencies with insights into these mediating roles, guiding targeted public health strategies to reduce COVID-19 prevalence in vulnerable populations and better prepare for future outbreaks.

## 2. Materials and Methods

### 2.1. Study Area

Located in Southern Nevada, Clark County contains the world-famous Las Vegas Strip and three incorporated cities (Las Vegas, North Las Vegas, and Henderson), as shown in Figure S1 of the Supplementary Material. As of 2022, Clark County had an estimated population of approximately 2.27 million residents living in 535 census tracts, with males comprising 50.2% of the population. Non-Hispanic Whites represented the largest racial group at 39.7%, and adults aged 25–49 accounted for the largest age group at 35.0%.

### 2.2. Data Sources

This study utilized geocoded and de-identified COVID-19 data processed by the Southern Nevada Health District, including confirmed cases, testing cases, full vaccination cases, and follow-up vaccination cases. In particular, we defined full vaccination as receiving two doses of the vaccine, while follow-up vaccination referred to an administered booster dose following full vaccination. The data were further aggregated at the census tract level to compute age-adjusted rates to ensure accurate and comprehensive analyses. The SVI, originally developed by the Agency for Toxic Substances and Disease Registry, is publicly available through the CDC website (https://www.atsdr.cdc.gov/place-health/php/svi/index.html, accessed on 1 July 2023). SVI data were derived from 16 five-year estimates of the American Community Survey. This study specifically used the 2020 SVI data to match our COVID-19 data collected up to 2022. The original SVI is calculated as a percentile rank based on the sum of the percentile percentages of these 16 variables, resulting in a value ranging from 0 to 1. However, interpreting associations between the SVI and COVID-19 outcomes using this scaled value poses challenges, as a one-unit increase in the SVI is not observable in any census tract. To address this issue and enhance the interpretability, we instead used the raw summation of the percentile percentages, allowing for more meaningful statistical interpretation of changes in COVID-19 measures relative to a unit change in the SVI. Thus, a one-unit increase in the SVI represents a relative increase in vulnerability rather than a tangible change within a population (e.g., one more person experiencing poverty) regarding communities’ vulnerability to distressing events.

This study is a population-based analysis utilizing aggregated data at the census tract level. As no individual-level data were used and no human subjects were involved, institutional review board approval was not required. All data sources are de-identified and comply with relevant ethical standards, ensuring that no ethical concerns are associated with the conduct of this study.

### 2.3. Statistical Analyses

We first utilized the simple mediation analysis to examine if the COVID-19 testing rate or full/follow-up vaccination rate was a significant mediator between the SVI and COVID-19 prevalence rate, respectively. Figure S2a in the Supplementary Material shows the conceptual diagram of the simple mediation model, which can be expressed as two linear regressions:

(1) $$M=i_{M}+aX+\sum_{i=1}^{6}\gamma_{i}{Covariate}_{i}+e_{M},$$

(2) $$Y=i_{Y}+c^{\prime }X+bM+\sum_{i=1}^{6}\gamma_{i}{Covariate}_{i}+e_{Y},$$

where M is the mediator from COVID-19 testing or vaccination rate, X is the SVI score as the main predictor, and Y indicates the COVID-19 prevalence rate. Both equations were adjusted by six covariates (i.e., inactive commuting, park deprivation, retail density, housing inadequacy, segregation, and population density). In addition, $i_{M}$ and $i_{Y}$ are regression intercepts, and a, b, $c^{\prime }$, and $\gamma_{i}$ are regression slopes. In particular, the coefficient c’ can be regarded as the direct effect to explain how two census tracts differed by one point on the SVI by c’ cases per 1000 people on the COVID-19 prevalence rate when the mediator was held constant. Additionally, multiplying the regression coefficients a and b can produce the indirect effect to explain as ab cases per 1000 people on the COVID-19 prevalence rate via the mediator when two census tracts differed by a point of the SVI. Thus, the total effect was denoted as c, which is equal to c’+ab, to quantify how much deviation occurred in the COVID-19 prevalence rate through a one-point increase in the SVI. The ordinary least squares method and its statistical inference estimated all coefficients of regression intercepts and slopes and their 95% confidence intervals (CIs). However, the confidence interval of the indirect effect was computed by the bootstrapping technique, where the resampling times were set to 5000. Hence, the significance of a mediator was determined by the 95% CI of the indirect effect strictly different from 0. Lastly, $e_{M}$ and $e_{Y}$ are error terms.

We further integrated the three mediators to examine a potential causal chain from the COVID-19 testing rate to the full vaccination rate, and subsequently to the follow-up vaccination rate. The Supplementary Material presents the corresponding conceptual diagram in Figure S2b. Thus, we conducted a serial mediation analysis to evaluate all mediators simultaneously within a unified framework, which involved estimating four linear regressions:

(3) $$M_{1}=i_{M_{1}}+a_{1}X+\sum_{i=1}^{6}\gamma_{i}{Covariate}_{i}+e_{M_{1}}$$

(4) $$M_{2}=i_{M_{2}}+a_{2}X+d_{21}M_{1}+\sum_{i=1}^{6}\gamma_{i}{Covariate}_{i}+e_{M_{2}}$$

(5) $$M_{3}=i_{M_{3}}+a_{3}X+d_{31}M_{1}+d_{32}M_{2}+\sum_{i=1}^{6}\gamma_{i}{Covariate}_{i}+e_{M_{3}}$$

(6) $$Y=i_{Y}+c^{\prime }X+b_{1}M_{1}+b_{2}M_{2}+b_{3}M_{3}+\sum_{i=1}^{6}\gamma_{i}{Covariate}_{i}+e_{Y}$$

where $M_{1}$, $M_{2}$, and $M_{3}$ equate to the COVID-19 testing rate, COVID-19 full vaccination rate, and COVID-19 follow-up vaccination rate, respectively. Similar to the simple mediation model, $i_{M_{1},}\;i_{M_{2}},\;i_{M_{3}}$, and $i_{Y}$ are regression intercepts, and $a_{1},a_{2,}\;a_{3},\;b_{1},\;b_{2},\;b_{3},\;d_{21},\;d_{31},$ and $d_{32}$ are regression slopes for estimating the multiple indirect, direct, and total effects. The methodology for computing the effects and unknown parameters of serial mediation analysis is the same as that of simple mediation analysis. However, its indirect effects depend on the three mediators. The computation for the indirect effects was performed by multiplying the values of the regression coefficients related to each step within the indirect effect pathway by the SVI toward the COVID-19 testing rate and sequentially to the COVID-19 vaccination rate. Thus, the total number of the indirect effects was cumulatively summed as the SVI’s total indirect effect, and the direct effects were summed for the total effect of the serial mediation model. Lastly, the error terms were labeled as $e_{M_{1}},\;e_{M_{2}\;},\;e_{M_{3}}$, and $e_{Y}$.

All model equations in the serial mediation analysis included the six covariates, but we conducted model selections on them to better generate significant total, direct, and indirect effects. In detail, we examined all combinations of covariates, ranging from none to six, to identify the best serial mediation model. Thus, the final serial mediation model contained three covariates regarding park deprivation, retail density, and segregation, which resulted in significant total, direct, and total indirect effects, with one significant individual indirect effect.

Model assumptions were diagnosed in all linear regressions, and the normality and variance homogeneity assumptions were violated in most regressions. Although these violations may not have been influential due to our large sample size, we still log-transformed all the COVID-19 measures to mitigate the impacts in data analyses. Thus, the influence of the SVI on COVID-19 measures was explained by its exponentiated estimated coefficient because all COVID-19 measures were log-transformed. The influence of mediators was explained as the percent change in the COVID-19 prevalence rate through the percent change in the mediator, since both the mediators and the COVID-19 prevalence rate were log-transformed. Lastly, because vaccination cases were assumed to be zero in 2020 when calculating vaccination rates, we conducted a sensitivity analysis using data accumulated from January 2021 to June 2022 to assess the robustness of our results.

SAS v9.4 (SAS Institute Inc., Cary, NC, USA) conducted all the mediation and serial mediation analyses via the PROCESS macro [38]. The significance level was set to 0.05.

## 3. Results

Table 1 summarizes the SVI and COVID-19 measures used in this study. The SVI ranged from 1.45 to 13.33, with an average of 7.79 (standard deviation (SD) = 2.55), and the COVID-19 prevalence rate ranged from 4.40 to 989.26 cases per 1000 people, with an average of 233.45 cases per 1000 people (SD = 78.58). The averages of the mediators were 1851.3 tests per 1000 people (SD = 757.59) for the COVID-19 testing rate, 611.49 cases per 1000 people (SD = 808.93) for the COVID-19 full vaccination rate, and 281.26 cases per 1000 people (SD = 124.75) for the COVID-19 follow-up vaccination rate. Their geographical distributions are shown in Figure S3 in the Supplementary Material.

The bivariate linear relationships are shown in Table 2, indicating that the SVI was significantly associated with all COVID-19 measures, with the strongest relationship found between the SVI and the COVID-19 follow-up vaccination rate, which had a correlation coefficient of −0.58 (95% CI = −0.63, −0.52). Among the COVID-19 measures, all testing and vaccination rates were positively associated with COVID-19 prevalence, with the strongest relationship found for the COVID-19 testing rate, which had a correlation coefficient of 0.78 (95% CI = 0.75, 0.81).

**Figure 1:**
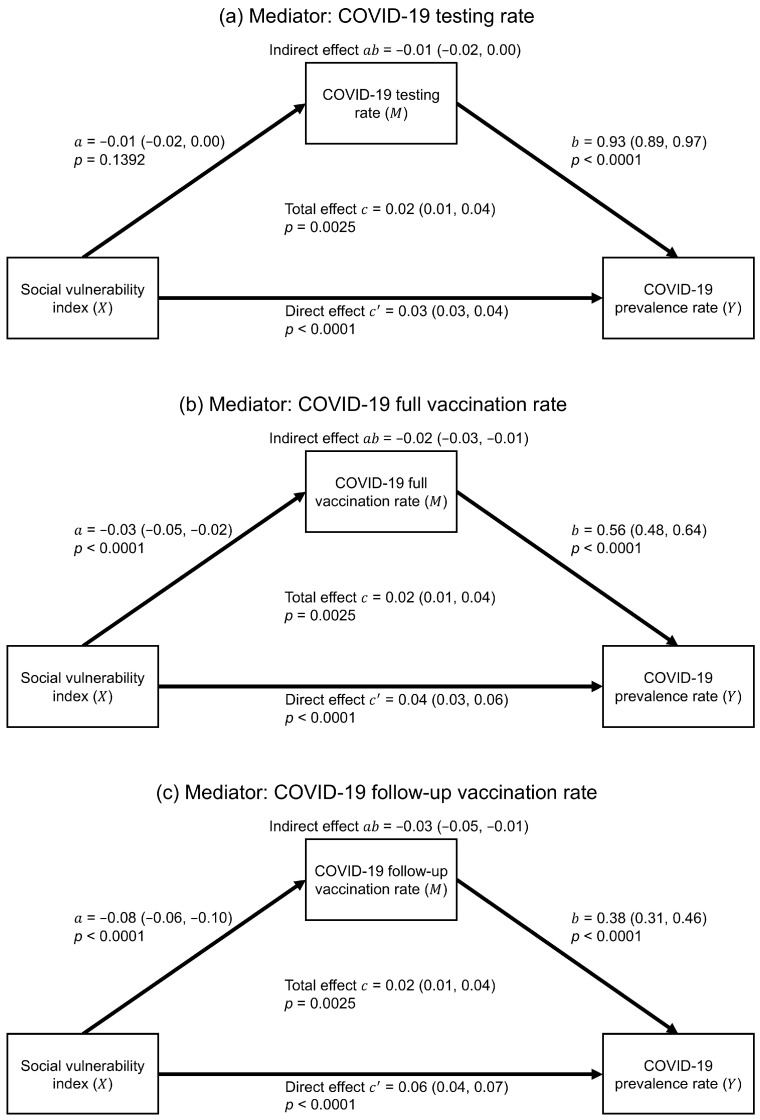
Estimated results of simple mediation analyses. Parentheses show 95% confidence intervals.

Figure 1 shows that, in the simple mediation analysis, the associations among the SVI, mediators, and COVID-19 prevalence were all statistically significant after adjusting for covariates. Among the three mediators, the SVI had the strongest association with the follow-up vaccination rate (a = −0.08; 95% CI = −0.06, −0.10; *p*-value < 0.0001). In addition, all mediators were significantly and positively associated with COVID-19 prevalence, with each 1% increase in the testing rate, full vaccination rate, and follow-up vaccination rate leading to an increase in the prevalence rate by 0.93% (95% CI = 0.89, 0.97; *p*-value < 0.0001), 0.56% (95% CI = 0.48, 0.64; *p*-value < 0.0001), and 0.38% (95% CI = 0.31, 0.46; *p*-value < 0.0001), respectively. The indirect effects of the SVI through the full vaccination rate (ab = −0.02; 95% CI = −0.03, −0.01) and the follow-up vaccination rate (ab = −0.03; 95% CI = −0.05, −0.01) to COVID-19 prevalence were significant. The direct effects (i.e., c’) revealed that the SVI significantly and positively influenced COVID-19 prevalence when either the testing rate or full/follow-up vaccination rate mediated their relationships. Lastly, the three simple mediation models resulted in an identical total effect (c = 0.02; 95% CI = 0.01, 0.04; *p*-value = 0.0025), indicating a significantly higher COVID-19 prevalence rate as the SVI increased, mediated by either the testing or vaccination rates.

**Figure 2:**
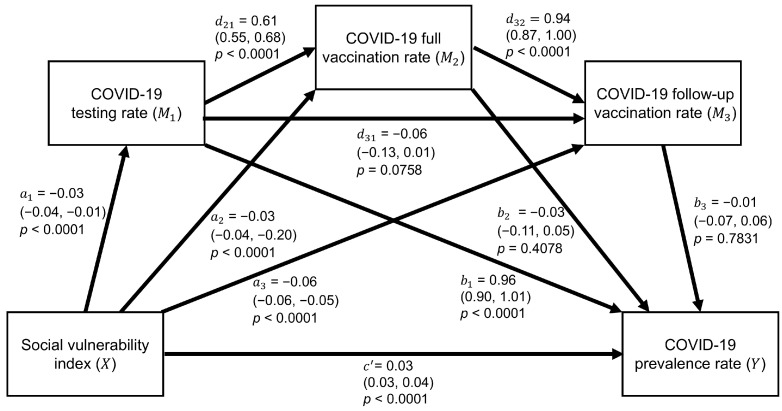
Estimated results of serial mediation analyses. Parentheses show 95% confidence intervals.

In the serial mediation analysis, seven of the ten associations were statistically significant, including the direct effect from the SVI to COVID-19 prevalence ($c^{\prime }$ = 0.03; 95% CI = 0.03, 0.04; *p*-value < 0.0001), as shown in Figure 2. The SVI was still significantly associated with the testing rate, full vaccination rate, and follow-up vaccination rate, all with a *p*-value < 0.0001. However, among the mediators, only the testing rate was still significantly associated with the prevalence rate ($b_{1}$= 0.96; 95% CI = 0.90, 1.01; *p*-value < 0.0001). Moreover, the full vaccination rate was significantly associated with the testing rate ($d_{21}$= 0.61; 95% CI = 0.55, 0.68; *p*-value < 0.0001) and the follow-up vaccination rate ($d_{32}$= 0.94; 95% CI = 0.87, 1.00; *p*-value < 0.0001), whereas no significant association was found between the testing and follow-up vaccination rates. Table 3 shows that the total indirect effect was significant (estimate = −0.02; 95% CI = −0.03, −0.002), while individual indirect effect 1 (i.e., SVI → COVID-19 testing rate → COVID-19 prevalence rate) was also significant (estimate = −0.02; 95% CI = −0.04, −0.01). This pathway included only the COVID-19 testing rate as a significant mediator for the SVI and COVID-19 prevalence rate. Finally, the total effect was positively significant (estimated coefficient = 0.01; 95% CI = 0.001, 0.02; *p*-value = 0.0354).

In the sensitivity analysis, Supplementary Material Table S1 shows that when rates were recalculated using data accumulated between January 2021 and June 2022, the indirect effects in the three simple mediation models remained consistent, with no change in statistical significance. In the serial mediation model, the 95% CI for the total indirect effect shifted slightly from (−0.03, 0.00) to (−0.05, −0.01), yielding a statistically significant mediation effect across the three mediators. Although the upper bound remained close to zero, the result is considered robust, particularly given the use of bootstrapping, which may introduce minor variations due to random number generation.

## 4. Discussion

This study is the first to examine COVID-19 testing and vaccination rates as mediators between the SVI and COVID-19 prevalence in the U.S. As hypothesized, higher SVI scores were associated with lower testing rates, leading to fewer reported cases of COVID-19, which aligns with other findings regarding communities with higher SVI scores being less likely to test for COVID-19 [7]. Similarly, higher SVI scores were negatively associated with both full and follow-up vaccination rates, resulting in more COVID-19 cases. Although no studies have analyzed vaccination rates as mediators between the SVI and COVID-19 prevalence, previous research indicated that vaccination uptake varies by SVI domains. Our serial mediation analysis showed a significant serial mediation effect from the SVI to COVID-19 prevalence. The pathway suggested that higher SVI scores were linked to lower testing rates, which were significantly associated with decreased vaccination rates, ultimately leading to increased COVID-19 prevalence. These findings emphasize the need to address access to testing and vaccination in socially vulnerable communities, as individuals living in these areas are more likely to be infected with COVID-19 and potentially other infectious diseases [39].

Simple mediation analyses revealed that the SVI was significantly and negatively associated with COVID-19 prevalence, with the follow-up vaccination rate showing the strongest association with higher COVID-19 cases. Previous studies have also found that factors related to the SVI, such as socioeconomic status, minority status, and low-income backgrounds, were significantly correlated with higher COVID-19 infection rates [40,41,42]. Research incorporating the SVI as a predictor further supports these findings, with studies in Florida [43], Texas [44], and Louisiana [45] showing that higher SVI scores were associated with increased COVID-19 cases and mortality, particularly among minority populations. These findings reinforce the evidence that socially vulnerable communities, as indicated by higher SVI scores, face a disproportionate burden of COVID-19.

The COVID-19 testing rate emerged as a significant mediator between the SVI and COVID-19 prevalence in both simple and serial mediation analyses, with high SVI census tracts experiencing lower testing rates and, consequently, fewer reported cases. Variability in the SVI was proportionally related to COVID-19 prevalence, even when controlling for testing, while the indirect effect suggested that underreporting and asymptomatic cases might lead to underestimation. Few studies have examined testing as a mediator, but this study offers valuable insights into its role in vulnerable communities. Supporting evidence includes findings from Illinois schools, where higher testing rates were linked to lower positivity [46]. Additionally, lower testing rates have been linked to higher SVI scores in ZIP codes across U.S. cities [7], reduced socioeconomic status from the SVI in Massachusetts [47], and increased SVI in census block groups in 30 cities [48]. While much of the literature focused on test positivity [49,50,51], variability in testing’s association with prevalence was evident. In Central Florida, lower testing rates correlated with higher hospitalizations [52]. Our findings underscore the critical role of testing as a mediator, as lower testing may lead to underreporting of COVID-19 cases, particularly due to unrecorded home testing and undetected asymptomatic infections.

Our study found that individuals in census tracts with higher SVI scores were less likely to receive full COVID-19 vaccinations or follow-up doses, increasing their risk of infection and reinfection. This aligns with other studies that reported lower vaccination rates in socially vulnerable areas [53,54], although one study observed higher vaccination rates in less vulnerable counties [55]. This study identified a direct link between vaccination rates and COVID-19 prevalence, with lower vaccination rates being associated with more cases. Similar trends were noted in previous studies showing lower hospitalization and fatality risks with higher vaccination rates [56,57]. These findings emphasize the critical role of vaccination in safeguarding vulnerable populations.

This study examined the theoretical implications by advancing the understanding of how COVID-19 affects socially vulnerable communities through a mediation framework. Specifically, we employed mediation analyses to examine the role of testing and vaccination rates as sequential mediators in the relationship between SVI and COVID-19 prevalence. Our findings contribute to the literature by demonstrating a serial mediation pathway in which higher social vulnerability leads to lower testing rates, which in turn are related to lower vaccination uptake, ultimately impacting COVID-19 prevalence. From a practical perspective, this study underscores the utility of the SVI not only as a tool for identifying at-risk communities but also for guiding resource allocation and developing culturally responsive health strategies to prioritize efforts that improve access to both testing and vaccination services in socially vulnerable populations, effectively reducing disease burden.

The variability in the significance of mediation effects across periods warrants explanation. In the sensitivity analysis, the total effect of SVI on COVID-19 prevalence was significant from January 2020 to June 2022, but not from January 2021 to June 2022. This shift likely reflects evolving pandemic conditions. In 2020, limited testing, absence of vaccines, and high uncertainty likely amplified the association between social vulnerability and COVID-19 outcomes. In contrast, the later period saw increased at-home testing, broader vaccine access, and behavioral changes, such as medical avoidance, which may have weakened the observable link between the SVI and reported cases. These changes underscore the importance of interpreting mediation results in context. We recommend that future studies account for policy changes, variant emergence, and testing/reporting practices that may influence such associations.

This study has some limitations, including the applicability of the SVI, which may not generalize well to states with varying census tract sizes. The inability of mediation analysis to establish causation between independent variables and linear relationships is also a limitation. Partial vaccination was excluded from this study due to multicollinearity, particularly in the serial mediation analysis. Additionally, model violations and a small total indirect effect coefficient raise concerns about reliability; however, small coefficients are typical in mediation analyses involving multiple mediators [58]. Prior studies have reported significantly small coefficients in similar contexts [59,60,61], and mediation analysis is sensitive to model assumptions as indirect effects increase [38,62]. Lastly, our analysis was limited to data from Clark County, Nevada. To date, we have found no prior studies that conducted similar mediation analyses in other areas. As such, the generalizability of our findings may be limited, and our results should be interpreted conservatively. Further research is needed to assess whether these mediation effects are consistent in other regions. Despite these challenges, our findings indicated that COVID-19 testing and vaccination rates remain important mediators in the relationship between the SVI and COVID-19 prevalence, particularly in resource-limited, socially vulnerable communities.

This study developed a prototype for identifying disparities in COVID-19 testing and vaccination rates across census tracts with varying levels of social vulnerability. Future research should consider stratified analyses by gender and race to further elucidate the intersectionality of social vulnerability and health outcomes. Such work could reveal whether certain subpopulations, such as racial minorities or specific gender groups in high-SVI areas, experience disproportionate barriers to healthcare access. These findings could inform more targeted and equitable public health interventions.

## 5. Conclusions

The inadequate response from national and state governments during the COVID-19 pandemic has significantly affected socially vulnerable communities, limiting their access to testing and vaccination sites. This reflects a broader pattern of neglect seen in socially vulnerable communities, as evidenced by past adverse events, such as Hurricane Katrina. This cycle of neglect may contribute to distrust in healthcare professionals, resulting in low vaccine uptake, as evidenced by only 60% of individuals in Nevada being fully vaccinated. To address these disparities, it is crucial to implement community-focused interventions and culturally sensitive educational programs for health policymakers. This study focused on a metropolitan area—further research should explore mediating factors related to disparities in rural populations.

## Figures and Tables

**Table 1:** Summary statistics of the social vulnerability index and COVID-19 measures (unit: per 1000 people).

Variable	Mean	SD	Min.	Q1	Median	Q3	Max.
Social vulnerability index	7.79	2.55	1.45	5.74	7.64	9.90	13.33
COVID-19 prevalence rate	233.45	78.58	4.40	193.29	224.66	260.02	989.26
COVID-19 testing rate	1851.30	757.59	49.86	1558.21	1756.14	1995.45	12,476.19
COVID-19 full vaccination rate	611.49	808.93	45.70	492.57	546.36	616.19	17,371.41
COVID-19 follow-up vaccination rate	281.26	124.75	0.00	213.89	260.92	323.84	1270.61

Abbreviations: SD = standard deviation; Q1 = first quartile; Q3 = third quartile.

**Table 2:** Pearson’s correlations and 95% confidence intervals among the social vulnerability index and log-transformed COVID-19 measures.

Variable	COVID-19 Prevalence Rate	COVID-19 Testing Rate	COVID-19 Full Vaccination Rate	COVID-19 Follow-Up Vaccination Rate
Social vulnerability index	−0.07 (−0.15, 0.02)	−0.26 (−0.33, −0.18)	−0.13 (−0.21, −0.04)	−0.58 (−0.63, −0.52)
COVID-19 prevalence rate		0.78 (0.75, 0.81)	0.31 (0.24, 0.39)	0.55 (0.48, 0.60)
COVID-19 testing rate			0.71 (0.66, 0.75)	0.72 (0.67, 0.75)
COVID-19 full vaccination rate				0.52 (0.46, 0.58)

**Table 3:** Serial mediation analysis of COVID-19 testing and vaccination rates as the mediator between the social vulnerability index and COVID-19 prevalence rate.

Path ^§^	Effect ^§§^	95% Confidence Interval	*p*-Value
Total effect (c)	0.01	(0.001, 0.02)	0.0354
Direct effect (c’)	0.03	(0.03, 0.04)	<0.0001
Total indirect effect	−0.02	(−0.03, −0.002)	-
Individual indirect effect 1	−0.02	(−0.04, −0.01)	-
Individual indirect effect 2	0.001	(−0.01, 0.007)	-
Individual indirect effect 3	0.001	(−0.01, 0.02)	-
Individual indirect effect 4	0.001	(−0.01, 0.004)	-
Individual indirect effect 5	−0.00	(−0.002, 0.004)	−
Individual indirect effect 6	0.0003	(−0.003, 0.01)	−
Individual indirect effect 7	0.0001	(−0.002, 0.01)	−

^§^ Individual indirect effect 1: SVI → COVID-19 testing rate → COVID-19 prevalence rate; individual indirect effect 2: SVI → COVID-19 full vaccination rate → COVID-19 prevalence rate; individual indirect effect 3: SVI → COVID-19 follow-up vaccination rate → COVID-19 prevalence rate; individual indirect effect 4: SVI → COVID-19 testing rate → COVID-19 full vaccination rate → COVID-19 prevalence rate; individual indirect effect 5: SVI → COVID-19 testing rate → COVID-19 follow-up vaccination rate → COVID-19 prevalence rate; individual indirect effect 6: SVI → COVID-19 full vaccination rate → COVID-19 follow-up vaccination rate → COVID-19 prevalence rate; individual indirect effect 7: SVI → COVID-19 testing rate → COVID-19 full vaccination rate → COVID-19 follow-up vaccination rate → COVID-19 prevalence rate. ^§§^ All effects were adjusted by covariates.

## Data Availability

The codes used in this study are available upon request from the corresponding author. The COVID-19 prevalence, testing, and vaccination rate data are not publicly available due to ethical and legal restrictions of the agency.

## Supplementary Materials

The following supporting information can be downloaded at: https://www.mdpi.com/article/10.3390/ijerph22070980/s1. Table S1: Sensitivity analysis of simple and serial mediation models; Figure S1: Census tract boundaries in Clark County and the Las Vegas metropolitan area; Figure S2: Conceptual diagrams of the (a) simple mediation model and (b) serial mediation model; Figure S3: The distribution of social vulnerability index and COVID-19 measures at the census tract level in Clark County, Nevada. All COVID-19 measures were age-adjusted. Gray areas indicate no data.
