# Supplementary material for: The Role of Testing and Vaccination in Mediating Social Vulnerability and COVID-19 Prevalence in Southern Nevada
Source: Int J Environ Res Public Health. 2025 Jun 21;22(7):980. doi: 10.3390/ijerph22070980 (PMC12294772; doi:10.3390/ijerph22070980)

Supplementary material

Figure S1. Census tract boundaries in Clark County and the Las Vegas metropolitan area.

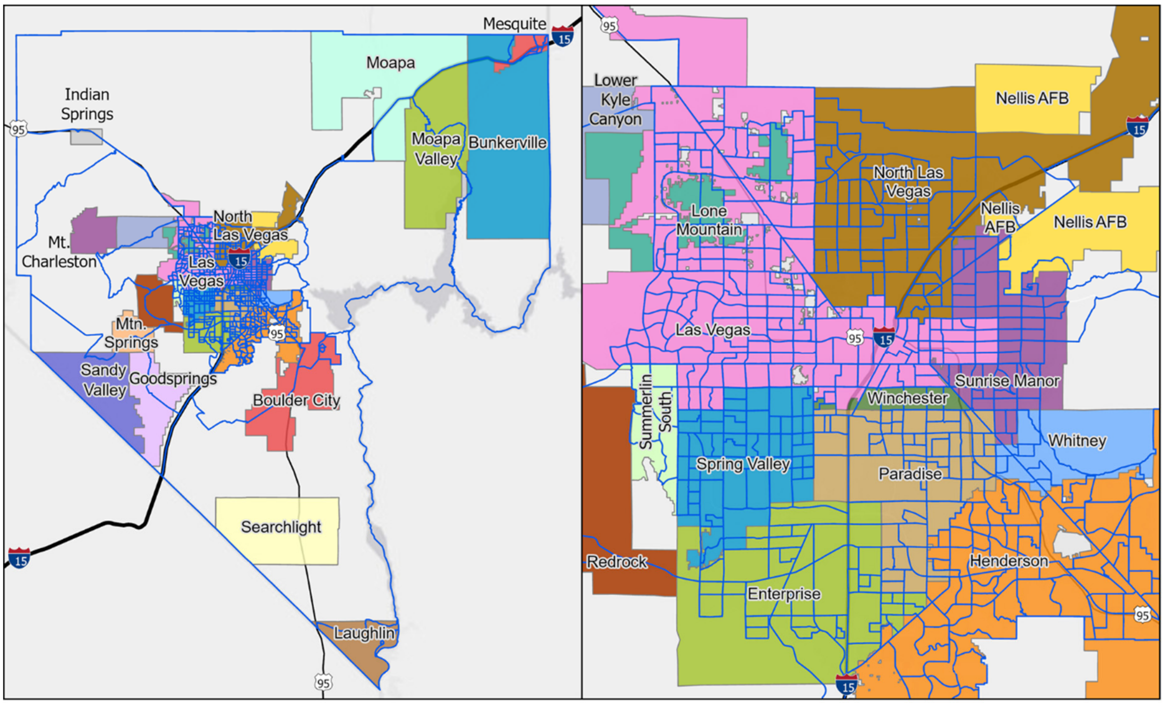

Table S1. Sensitivity analysis of simple and serial mediation models.

	2020/01-2022/06				2021/01-2022/06			
**Parameter**	**Est**	**95% CI**		**P-value**	**Est**	**95% CI**		**P-value**
Mediator: COVID-19 testing rate								
a	-0.01	-0.03	0.00	0.1392	-0.02	-0.03	0.00	0.0165
b	0.93	0.89	0.97	<0.0001	0.88	0.84	0.91	<0.0001
c' (direct effect)	0.04	0.03	0.04	<0.0001	0.02	0.02	0.03	<0.0001
indirect effect	-0.01	-0.03	0.00		-0.02	-0.03	0.00	
total effect	0.02	0.01	0.04	0.0025	0.01	-0.01	0.02	0.4977
Mediator: COVID-19 full vaccination rate								
a	-0.03	-0.05	-0.02	<0.0001	-0.03	-0.05	-0.02	<0.0001
b	0.56	0.48	0.64	<0.0001	0.51	0.43	0.58	<0.0001
c' (direct effect)	0.04	0.03	0.06	<0.0001	0.02	0.01	0.04	0.0012
indirect effect	-0.02	-0.03	-0.01	---	-0.02	-0.03	-0.01	---
total effect	0.02	0.01	0.04	0.0025	0.01	-0.01	0.02	0.4977
Mediator: COVID-19 follow-up vaccination rate								

a	-0.08	-0.10	-0.06	<0.0001	-0.08	-0.10	-0.06	<0.0001
b	0.38	0.31	0.46	<0.0001	0.36	0.28	0.43	<0.0001
c' (direct effect)	0.06	0.04	0.07	<0.0001	0.03	0.02	0.05	<0.0001
indirect effect	-0.03	-0.05	-0.01	---	-0.03	-0.05	-0.01	---
total effect	0.02	0.01	0.04	0.0025	0.01	-0.01	0.02	0.4977
Mediator: COVID-19 testing rate + COVID-19 full vaccination rate + COVID-19 follow-up vaccination rate								
a_1	-0.03	-0.04	-0.01	<0.0001	-0.03	-0.04	-0.02	<0.0001
a_2	-0.03	-0.04	-0.02	<0.0001	-0.03	-0.04	-0.02	<0.0001
a_3	-0.06	-0.06	-0.05	<0.0001	-0.06	-0.07	-0.05	<0.0001
b_1	0.96	0.90	1.01	<0.0001	0.90	0.85	0.94	<0.0001
b_2	-0.03	-0.11	0.05	0.4078	-0.11	-0.18	-0.03	0.0065
b_3	-0.01	-0.07	0.06	0.7831	0.08	0.02	0.14	0.0121
d_{21}	0.61	0.55	0.68	<0.0001	0.57	0.50	0.64	<0.0001
d_{31}	-0.06	-0.13	0.01	0.0753	-0.11	-0.17	-0.04	0.0008
d_{32}	0.94	0.87	1.00	<0.0001	0.96	0.90	1.03	<0.0001
c' (direct effect)	0.03	0.03	0.04	<0.0001	0.03	0.02	0.03	<0.0001
total indirect effect	-0.02	-0.03	0.00	---	-0.03	-0.05	-0.01	---
total effect	0.01	0.00	0.02	0.0354	-0.01	-0.02	0.00	0.1980

Figure S2. Conceptual diagrams of the (a) simple mediation model and (b) serial mediation model.

(a)

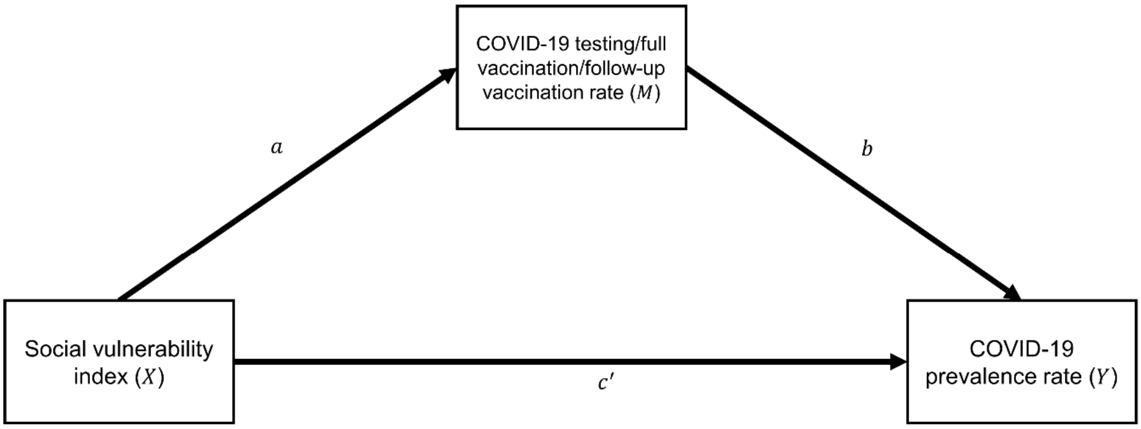

(b)

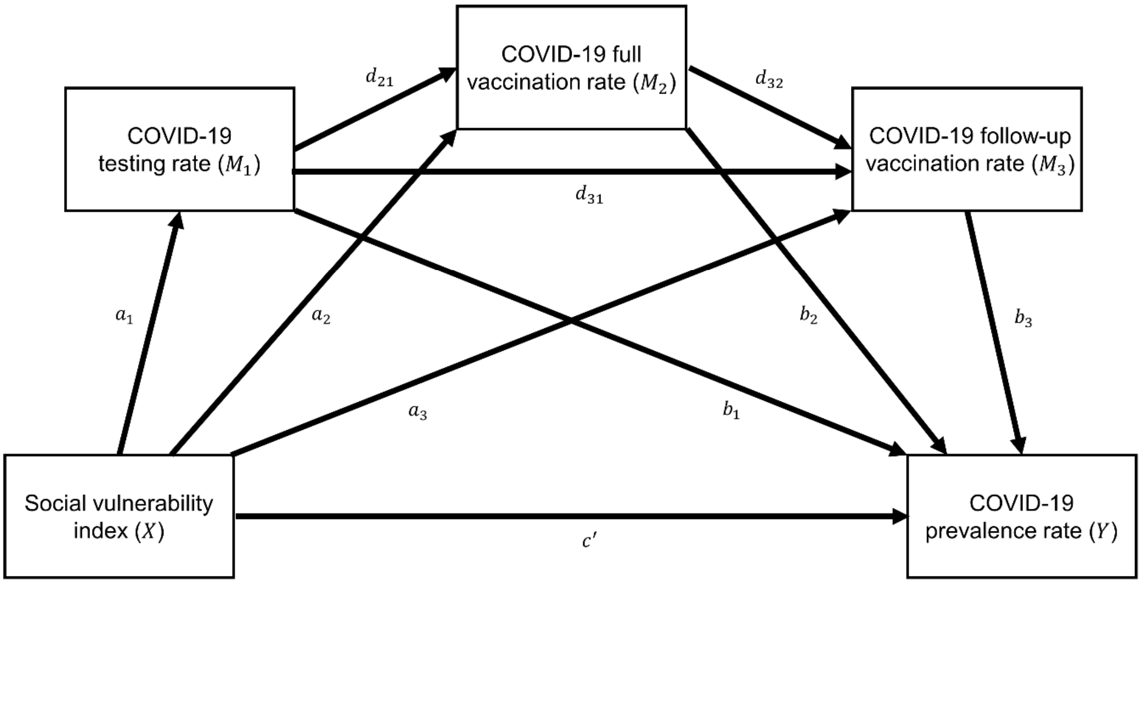

Figure S3. The distribution of social vulnerability index and COVID-19 measures at the census tract level in Clark County, Nevada. All COVID-19 measures were age-adjusted. Gray areas indicate no data.

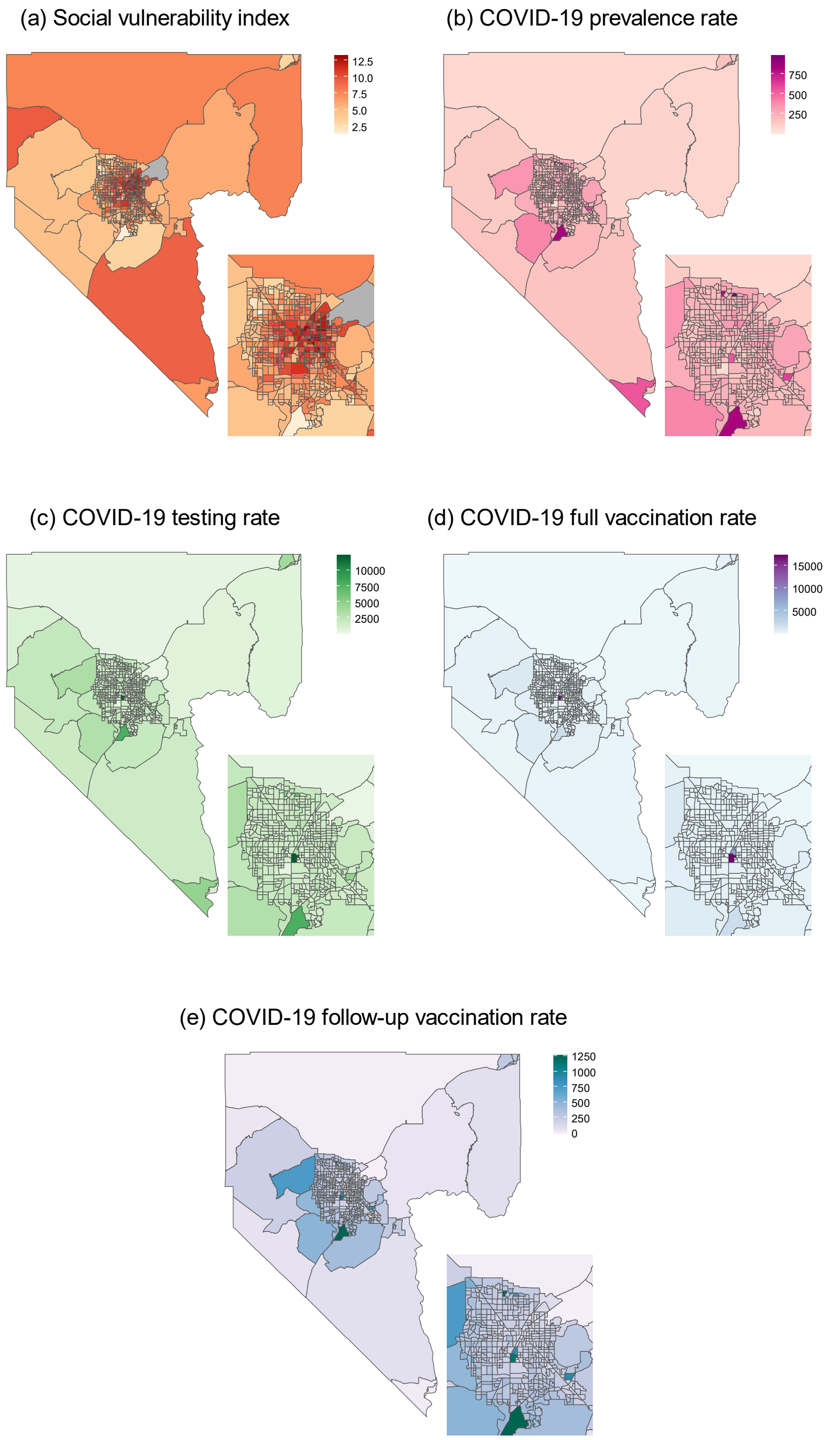

Supplement: Supplementary file 1 [file ijerph-22-00980-s001.zip › ijerph-3554268-supplementary.pdf]
